# Systematic metabolic engineering of *Escherichia coli* for high-level production of pseudouridine via pathway optimization and precursor enhancement

**DOI:** 10.1016/j.synbio.2026.03.013

**Published:** 2026-04-06

**Authors:** Jing Song, Wei Shen, Yuanyuan Xia, Li Zhou, Yingjun Zhou, Haiquan Yang, Xianzhong Chen

**Affiliations:** a School of Biotechnology and Key Laboratory of Carbohydrate Chemistry and Biotechnology of Ministry of Education, Jiangnan University, Wuxi, 214122, China; b School of Biotechnology and Key Laboratory of Industrial Biotechnology of Ministry of Education, Jiangnan University, Wuxi, 214122, China; c Xiangya School of Pharmaceutical Sciences, Central South University, Changsha, 410013, China

**Keywords:** Rational reconstruction, Pathway optimization, Enhanced production, *Escherichia coli*, Pseudouridine

## Abstract

Pseudouridine, the C5-ribose epimer of uridine with significant biological functions and clinical applications, was efficiently produced through systematic metabolic strategy of *Escherichia coli* in this study. Initial overexpression of pseudouridine-5-phosphate glycosylase gene *psuG* and alkaline phosphatase gene *YjjG* in *E*. *coli* pRSFDuet-1-*YjjG*-*psuG* yielded 0.43 g L^−1^ pseudouridine, which increased 8.56-fold with 5 g L^−1^ uridine supplementation. Subsequent deletion of *thrA*, *psuT*, *argF*, and *pepA* enhanced titer by 1.29-fold in *E*. *coli* Δ*thrA*Δ*psuT*Δ*argF*Δ*pepA* pRSFDuet-1-*YjjG*-*psuG*, while ribonucleoside hydrolase gene *rihA* overexpression boosted titer to 5.57 g L^−1^. Further optimization through deleting the uridine kinase gene *udk* and overexpressing the ribokinase gene *rbsK* in strain *E*. *coli* Δ*thrA*Δ*psuT*Δ*argF*Δ*pepA*Δ*udk*Δ*udp*Δ*ppnp* pRSFDuet-1-*YjjG*-*psuG* pCDFDuet-1-*rihA-rbsK* achieved 6.23 g L^−1^ pseudouridine, increasing to 11.34 g L^−1^ with two-stage uridine feeding. Fed-batch fermentation in a 5-L bioreactor yielded a record 102.2 g L^−1^ pseudouridine. This work provides an efficient and scalable bioprocess for industrial pseudouridine manufacturing to meet the growing demands of mRNA-based applications.

## Graphical abstract

**Figure:**
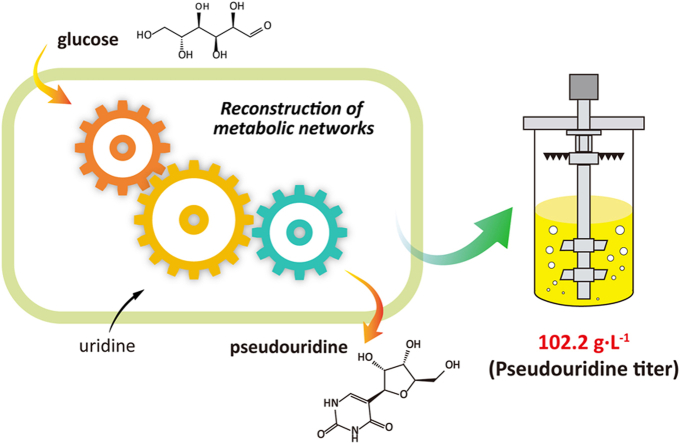

## 1 Introduction

Pseudouridine, a structural isomer of uridine, differs in its glycosidic bond configuration where the base-ribose linkage transitions from an N–C to a C–C bond, accompanied by a 180° rotation of the nucleobase about the N3–C6 axis [1]. As the first discovered and most prevalent RNA modification, pseudouridine is often termed the “fifth nucleoside” due to its capacity to participate in base pairing [2,3]. This modification confers unique structural and functional characteristics to RNA molecules [4], notably enhancing mRNA stability while substantially reducing immunogenicity [5], and moderately improving translational efficiency [6]. Leveraging the unique properties of pseudouridine, an RNA Codon Expansion (RCE) strategy was developed to utilize biorthogonal Ψ-based codons (ΨGA, ΨAA, ΨAG) for site-specific incorporation of non-canonical amino acids in mammalian systems. This approach enabled precise decoding of engineered mRNA transcripts bearing these modified codons [7]. These properties have proven crucial in COVID-19 mRNA vaccine development, where pseudouridine incorporation enhances both safety and efficacy by minimizing vaccine immunogenicity. The clinical applications of pseudouridine extend beyond vaccinology. As an RNA-specific metabolite excreted exclusively through renal pathways, it serves as a valuable biomarker for tumor monitoring and renal disease management [8]. Furthermore, its dual capacity to boost protein expression while reducing immunogenicity has established pseudouridine as an essential component in mRNA vaccine manufacturing [9]. This growing importance is reflected in the expanding market demand, particularly with advancing mRNA vaccine technologies. Meanwhile, pseudouridine is a promising candidate for synthesizing highly effective RNA-based biopesticides, paving the way for precise, programmable, and environmentally sustainable agricultural technologies.

Current pseudouridine production encompasses three primary methodologies: chemical synthesis, enzymatic methods, and microbial biosynthesis using engineered strains. The inaugural chemical synthesis of β-pseudouridine was achieved via condensation of 2,3,5-tri-*O*-benzoyl-d-ribofuranose with lithium 2,4-dimethoxy-pyrimidine, albeit with modest efficiency (2% yield) [10]. Subsequent methodological refinements have enhanced production metrics, yet persistent issues including step complexity, reagent toxicity, and environmental impact underscore the need for more sustainable synthetic strategies. Alice et al. pioneered the enzymatic synthesis of pseudouridine using uracil and ribose 5′-phosphate as substrates, where pseudouridine-5′-phosphate was first synthesized via pseudouridine 5′-phosphate glycosylase (PsuG) catalysis followed by alkaline phosphatase (phoA)-mediated dephosphorylation to produce pseudouridine [11]. While representing the first enzymatic route, this method faced challenges in enzyme concentration and activity control leading to inconsistent reaction times and substrate conversion rates, coupled with high enzyme preparation costs, prompting the development of engineered strains for more efficient production. Zhou et al. employed *E*. *coli* MG1655 as the parental strain, overexpressing key pathway enzymes while eliminating competing pathways through targeted gene knockouts, ultimately obtaining the high-yielding strain *E*. *coli* PSU5(pET30yjjGRspsuG) that produced 7.9 g L^−1^ pseudouridine in a 5-L fermenter [12]. Meanwhile, *E*. *coli* MG1655 was engineered through pTrc99a-mediated overexpression of the pseudouridine-5-phosphate glycosylase gene *psuG* and alkaline phosphatase gene *YjjG* (with RBS optimization), enhanced uracil uptake via the membrane transporter gene *UraA* overexpression, and knocking out the glucose-6-phosphate isomerase gene *pgi*, yielding strain *E*. *coli* G2 that achieved 27.5 g L^−1^ pseudouridine titer using glucose and 30 g L^−1^ uracil supplementation [13]. These engineered strains enable rapid glucose-to-pseudouridine conversion under mild conditions with stable yields, establishing a viable industrial-scale production platform with significant application potential.

**Fig. 1:**
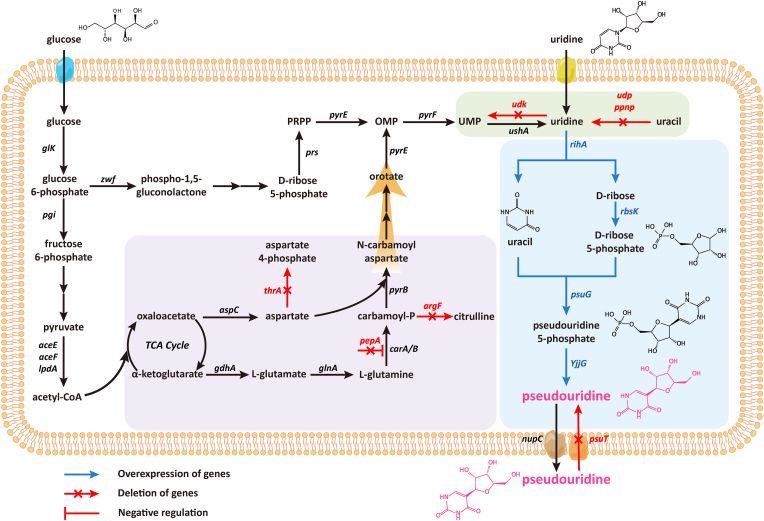
The pathway of efficiently synthesizing pseudouridine in *E*. *coli* via metabolic engineering. OMP, orotidine-5P; PRPP, 5-phospho-α-d-ribose 1 diphosphate; UMP, Uridine 5′-monophosphate. *glk*, glucokinase gene; *pgi*, glucose-6-phosphate isomerase gene; *aceE*, pyruvate dehydrogenase gene; *aceF*, dihydrolipoamide acetyltransferase; *lpdA*, dihydrolipoamide dehydrogenase; *aspC*, aspartate aminotransferase gene; *gdhA*, glutamate dehydrogenase gene; *glnA*, glutamine synthetase gene; *zwf*, NADP(+)-dependent glucose-6-phosphate dehydrogenase gene; *prs*, ribose-phosphate pyrophosphokinase gene; *pyrB*, aspartate carbamoyltransferase catalytic subunit gene; *pyrE*, orotate phosphoribosyltransferase gene; *pyrF*, orotidine-5′-phosphate decarboxylase gene; *udk*, uridine kinase gene; *udp*, uridine phosphorylase gene; *ppnp*, purine/pyrimidine-nucleoside phosphorylase gene; *ushA*, 5′-nucleotidase/UDP-sugar diphosphatase gene; *thrA*, fused aspartate kinase/homoserine dehydrogenase gene; *rihA*, pyrimidine-specific ribonucleoside hydrolase gene; *rbsK*, ribokinase gene; *psuG*, pseudouridylate synthase gene; *YjjG*, ΨMP-specific phosphatase gene; *nupC*, nucleoside:H(+) symporter gene; *psuT*, putative pseudouridine transporter gene; *pepA*, aminopeptidase A/I gene; *carA/B*, carbamoyl-phosphate synthetase gene; *argF*, ornithine carbamoyltransferase gene.

In this study, we constructed a recombinant *E*. *coli* for high-efficiency pseudouridine production through systematic metabolic engineering, achieving substantial yield improvement (Fig. 1). The systematic metabolic engineering strategy employed involved multiple key modifications: (i) overexpression of the key enzyme genes *psuG*, *YjjG*, *rihA*, and *rbsK* to optimize the pseudouridine biosynthetic pathway, (ii) deletion of the key genes *thrA*, *argF*, and *pepA* to enhance intracellular uracil accumulation, (iii) elimination of the gene *psuT* to prevent pseudouridine reuptake, (iv) removing the key genes *udk*, *udp*, and *ppnp* to block uridine degradation and increase precursor availability. The engineered strain demonstrated exceptional pseudouridine production capacity during fed-batch fermentation in a 5-L bioreactor.

## 2 Materials and methods

### 2.1 Strains and plasmids

*E*. *coli* BL21 (DE3) served as both the gene cloning host and initial pseudouridine production strain. The plasmids pRSFDuet-1 and pCDFDuet-1 were utilized for the key enzyme gene overexpression in *E*. *coli* BL21 (DE3), while the CRISPR-Cas9 system plasmids pCas and pTargetF facilitated gene knockouts. All strains and plasmids employed or constructed in this study were detailed in Table S1 and Table S2, respectively. Primers’ synthesis was performed by GenScript Biotechnology Corporation (Suzhou, China), with sequences provided in Table S3. Gene IDs of key genes (*YjjG*, *psuG*, *rihA*, and *rbsK*) were shown in Table S4.

### 2.2 Reagents

Peptone and yeast extract were purchased from Angel Yeast Co., Ltd. (Yichang, China). Glycerol, KH_2_PO_4_, K_2_HPO_4_, NaCl, methanol, and phosphoric acid were obtained from Sinopharm Chemical Reagent Co., Ltd. (Shanghai, China). l-arabinose was purchased from Aladdin Biochemical Technology Co., Ltd. (Shanghai, China). Uridine, pseudouridine, kanamycin, streptomycin, spectinomycin, and isopropyl β-d-1-thiogalactopyranoside (IPTG) were purchased from Macklin Biochemical Technology Co., Ltd. (Shanghai, China). Uracil was purchased from Beijing Solarbio Science & Technology Co., Ltd. (Beijing, China). Genomic DNA extraction kits, plasmid extraction kits, gel purification kits, column purification kits, and 2 × Taq Master Mix were obtained from Jiangsu Cowin Biotech Co., Ltd. (Taizhou, China). Ex Taq DNA Polymerase was purchased from Takara Bio Inc. (Beijing, China).

### 2.3 Media and culture conditions

The culture media compositions were as follows: LB medium (10 g L^−1^ peptone, 5 g L^−1^ yeast extract, 10 g L^−1^ NaCl), TB medium (12 g L^−1^ peptone, 24 g L^−1^ yeast extract, 4 mL L^−1^ glycerol, 2.31 g L^−1^ KH_2_PO_4_, 12.54 g L^−1^ K_2_HPO_4_), and bioreactor fermentation medium (12 g L^−1^ peptone, 24 g L^−1^ yeast extract, 4 mL L^−1^ glycerol, 2.31 g L^−1^ KH_2_PO_4_, 12.54 g L^−1^ K_2_HPO_4_), with the latter prepared in a 2-L batches. For recombinant *E*. *coli* cultivation, single colonies were first grown on solid LB medium at 37 °C for 10 h, then inoculated into 20 mL LB medium (in 100-mL shaking flask) and cultured at 37 °C and 200 rpm for 9 h. Subsequently, seed culture was transferred at 2% (v/v) inoculum into 50 mL TB medium (in 250-mL shaking flask) and grown under identical conditions until OD_600_ reached 1.0‒1.2, followed by temperature reduction to 25 °C and induction with 1.0 mM IPTG.

### 2.4 Construction of recombinant plasmids

The construction of pRSFDuet-1-*YjjG* plasmid served as an illustrative example. The *YjjG* gene was PCR-amplified from *E*. *coli* BL21 genomic DNA using primers *YjjG*-FW and *YjjG*-RS under the following cycling conditions: initial denaturation at 98 °C for 3 min; 30 cycles of 98 °C for 15 s (denaturation), 55 °C for 30 s (annealing), and 72 °C for extension (duration determined by amplicon length at 1 kb min^−1^); followed by final extension at 72 °C for 10 min. Both the amplified fragment and the pRSFDuet-1 plasmid were subjected to double-digestion with *Eco*RI and *Hin*dIII, purified using a column purification kit, and ligated with Ligation Solution I. The ligation mixture was transformed into chemically competent *E*. *coli* BL21 (DE3) cells and plated on LB agar containing 50 mg L^−1^ kanamycin, followed by 12 h incubation at 37 °C. Selected single colonies were cultured in LB medium at 37 °C and 200 rpm for 12 h for plasmid extraction, with Successful recombinants verified through *Eco*RI and *Hin*dIII double digestion and sequencing.

### 2.5 Gene knockout

This study employed the CRISPR/Cas9 system for *E*. *coli* genome engineering [14], utilizing plasmids pCas and pTargetF. The pTargetF plasmid, containing sgRNA fragments targeting specific gene, was amplified via PCR using primers incorporating the designed sgRNA sequences. Following *Dpn* I digestion of the template plasmid, the product was transformed into chemically competent *E*. *coli* BL21 cells, and plated on LB ager containing 100 mg·L^−1^ spectinomycin at 37 °C for 12 h. Positive recombinant plasmids (pTargetF-sgRNA) were identified through colony selection, plasmid extraction, and sequencing. Approximately 500 bp upstream and downstream homologous arms were PCR-amplified from the *E*. *coli* BL21 genome DNA and assembled into a recombinant template fragment by overlap extension PCR. Concurrently, pCas was transformed into *E*. *coli* BL21, plated on LB ager containing 50 mg L^−1^ kanamycin, and incubated at 30 °C for 12 h. Selected colonies were cultured in 20 mL LB medium (in 100-mL shaking flask) at 30 °C and 200 rpm for 10 h, then subcultured (1% inoculum) in 50 mL LB medium (in 250-mL shaking flask) until OD_600_ reached 0.2. Cas9 expression was induced with 1.5 mM l-arabinose until the OD_600_ reached 0.6, followed by electrocompetent cells preparation. The pTargetF-sgRNA plasmid and recombinant template fragment were co-transformed via electroporation, recovered in LB medium (30 °C, 200 rpm, 2 h), and plated on LB ager containing 50 mg L^−1^ kanamycin and 100 mg·L^−1^ spectinomycin at 30 °C for 12 h. Successful knockouts were verified by colony PCR, and positive colonies were cultured in 1 mL LB medium containing 1 mM IPTG (30 °C, 200 rpm, 2 h) before plating on kanamycin plates to eliminate pTargetF-sgRNA. Final knockout colonies were obtained by culturing at 42 °C for 12 h to remove pCas.

### 2.6 Production of pseudouridine in a 5-L bioreactor

The engineered strain was first pre-cultured in 50 mL of LB medium (500 mL shaking flask) at 37 °C and 200 rpm. When the cell density OD_600_ reached 1.8, a 5% (v/v) inoculum was transferred into a 5-L bioreactor containing 2 L of initial medium. The fermentation was conducted with an aeration rate of 1.5 vvm. The pH was automatically maintained at 7.0 by the addition of 30% (v/v) ammonium hydroxide. Dissolved oxygen (DO) was controlled between 30% and 50% through agitation-speed coupling. After the initial glucose was depleted, indicated by a DO rebound, a concentrated glucose solution (500 g L^−1^) was supplemented to maintain the residual glucose concentration below 3 g L^−1^. At 8 h of cultivation, the temperature was shifted from 37 °C to 25 °C, and IPTG was added to a final concentration of 0.8 mM to induce expression. Simultaneously, a uridine feeding strategy was initiated. For the condition targeting a final uridine concentration of 70 g L^−1^, feeding was performed as follows: from 8 to 20 h with a 300 g·L^−1^ solution at 20 mL h^−1^, from 20 to 50 h at 6 mL h^−1^, and from 50 to 54 h with a 250 g·L^−1^ solution at 20 mL h^−1^. For the 120 g L^−1^ final uridine condition, the feeding profile was: 8–20 h with a 350 g·L^−1^ solution at 20 mL h^−1^, 20–36 h at 6.25 mL h^−1^, 36–40 h at 15 mL h^−1^, and 40–60 h with a 333.3 g·L^−1^ solution at 20 mL h^−1^.

### 2.7 Detection of pseudouridine

Pseudouridine, uracil, and uridine concentrations were quantified by HPLC using a Diamonsil 5 μm C18 column (250 × 4.6 mm) with the following parameters: mobile phase consisting of 5% methanol:water (95:5, v:v) adjusted to pH 3.0 with phosphoric acid, flow rate maintained at 1 mL min^−1^, column temperature set to 30 °C, detection wavelength fixed at 260 nm, and injection volume of 10 μL.

### 2.8 Statistical analysis

All assays were carried out with three biological replicates, and the resulting data are reported as mean ± standard deviation. A two-tailed Student's *t*-test was employed to evaluate statistical differences, and *p* < 0.05 was regarded as the threshold for significance.

## 3 Results and discussion

### 3.1 Enhancing pseudouridine synthesis via overexpression of genes psuG and YjjG

**Fig. 2:**
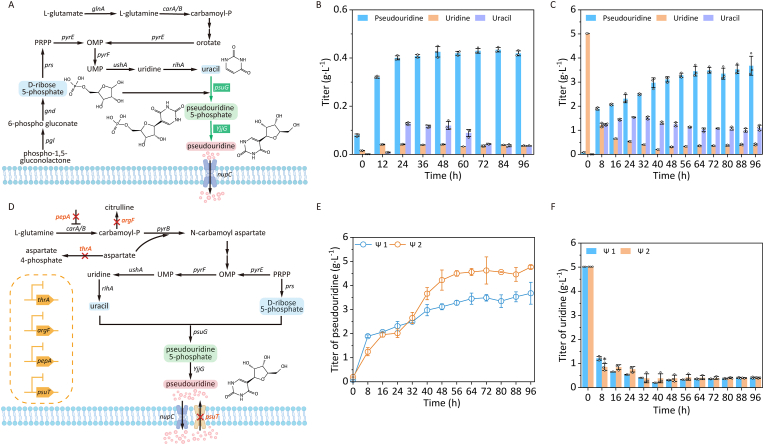
Enhanced the accumulation of pseudouridine in *E*. *coli* via overexpressing genes *psuG* and *YjjG*, enhancing intracellular uridine biosynthesis, and inhibiting pseudouridine transport. A, Overexpression of genes *psuG* and *YjjG* enhanced the accumulation of pseudouridine in *E*. *coli*. OMP, orotidine-5P; PRPP, 5-phospho-α-d-ribose 1 diphosphate; UMP, Uridine 5′-monophosphate; *pgl*, 6-phosphogluconolactonase gene; *gnd*, 6-phosphogluconate dehydrogenase gene; *prs*, ribose-phosphate pyrophosphokinase gene; *pyrE*, orotate phosphoribosyltransferase gene; *pyrF*, orotidine-5′-phosphate decarboxylase gene; *ushA*, 5′-nucleotidase/UDP-sugar diphosphatase gene; *rihA*, pyrimidine-specific ribonucleoside hydrolase gene; *glnA*, glutamine synthetase gene; *psuG*, pseudouridylate synthase gene; *YjjG*, ΨMP-specific phosphatase gene; *nupC*, nucleoside:H(+) symporter gene; *carA/B*, carbamoyl-phosphate synthetase gene. B, Production of pseudouridine in *E*. *coli* pRSFDuet-1-*YjjG*-*psuG* without adding uridine. C, Production of pseudouridine in *E*. *coli* pRSFDuet-1-*YjjG*-*psuG* by adding 5 g L^−1^ uridine. D, Effect of enhancing extracellular uridine biosynthesis and inhibiting pseudouridine transport on pseudouridine production. OMP, orotidine-5P; PRPP, 5-phospho-α-d-ribose 1 diphosphate; UMP, Uridine 5′-monophosphate; *prs*, ribose-phosphate pyrophosphokinase gene; *pyrE*, orotate phosphoribosyltransferase gene; *pyrF*, orotidine-5′-phosphate decarboxylase gene; *pyrB*, aspartate carbamoyltransferase catalytic subunit gene; *ushA*, 5′-nucleotidase/UDP-sugar diphosphatase gene; *rihA*, pyrimidine-specific ribonucleoside hydrolase gene; *psuG*, pseudouridylate synthase gene; *YjjG*, ΨMP-specific phosphatase gene; *thrA*, fused aspartate kinase/homoserine dehydrogenase gene; *nupC*, nucleoside:H(+) symporter gene; *psuT*, putative pseudouridine transporter gene; *pepA*, aminopeptidase A/I gene; *carA/B*, carbamoyl-phosphate synthetase gene; *argF*, ornithine carbamoyltransferase gene. E, Titer of pseudouridine. F, Titer of uridine. Ψ1, *E*. *coli* pRSFDuet-1-*YjjG*-*psuG*; Ψ2, *E*. *coli* Δ*thrA*Δ*psuT*Δ*argF*Δ*pepA* pRSFDuet-1-*YjjG*-*psuG*. No ∗, *p* > 0.05; ∗, *p* < 0.05.

*E*. *coli* naturally possesses the essential enzymes for pseudouridine synthesis, including pseudouridine-5-phosphate glycosylase (PsuG), which catalyzes the conversion of d-ribose and uracil to pseudouridine-5-phosphate, subsequently dephosphorylated by alkaline phosphatase YjjG to yield pseudouridine [15]. However, wild-type *E*. *coli* produced undetectably low pseudouridine levels. In this study, *E*. *coli* BL21 was engineered to enhance pseudouridine accumulation by overexpressing genes *psuG* and *YjjG*. The recombinant strain *E*. *coli* pRSFDuet-1-*YjjG*-*psuG* was constructed by transforming the co-expression plasmid pRSFDuet-1-*YjjG*-*psuG* into *E*. *coli* BL21 (Fig. 2A). *E*. *coli* pRSFDuet-1-*YjjG*-*psuG* achieved 0.43 g L^−1^ pseudouridine at 84 h (Fig. 2B), while uracil concentration peaked at 0.13 g L^−1^ (24 h) before declining to <0.05 g L^−1^ by 72 h; uridine remained consistently low (<0.05 g L^−1^). During the *de novo* synthesis of pseudouridine facilitated by *E*. *coli* pRSFDuet-1-*YjjG*-*psuG*, the specific pseudouridine production rate peaked in the early exponential growth phase. As the specific growth rate declined sharply, the specific pseudouridine productivity rate decreased correspondingly and approached near-zero levels (Fig. S1). These observations suggest that *de novo* pseudouridine synthesis was tightly coupled with active cellular metabolism. Cells in the exponential phase exhibited the most robust metabolic activity, thereby supporting the highest efficiency of product synthesis. Upon transition into the stationary phase, overall metabolic activity declined, leading to a concomitant reduction in biosynthetic capacity. To overcome limitations posed by uracil's poor aqueous solubility and ribose-5-phosphate's high cost [5,16] g·L^−1^ uridine was supplemented into fermentation medium, resulting in 3.68 g L^−1^ pseudouridine at 96 h (8.56-fold increase versus control) (Fig. 2C). Following uridine addition, uracil peaked at 1.54 g L^−1^ (24 h), gradually decreasing to 1.11 g L^−1^ (96 h), while uridine declined steadily, stabilizing at 0.40 g L^−1^ by 96 h. Upon uridine supplementation, the specific growth rate remained low during the initial phase, while the specific pseudouridine productivity rate increased and reached a peak. This phenomenon can be attributed to the preferential channeling of exogenous uridine into pseudouridine biosynthesis rather than biomass formation, leading to a transient suppression of early-stage growth. As uridine was depleted to a threshold level, both cell density and the specific growth rate recovered, culminating in maximum growth rate during the mid-phase. This shift indicated alleviation of the growth inhibition caused by high uridine availability, accompanied by a metabolic transition toward active cellular proliferation (Fig. S2). Multiple studies have demonstrated the critical role of ΨMP glycosylase and ΨMP phosphatase in constructing efficient pseudouridine biosynthesis pathways in *E*. *coli*. For example, Zhou et al. identified *RspsuG* (a pseudouridine-5′-phosphate glycosylase from *Rhizobium* sp. CF142) and *YjjG* (a ΨMP-specific phosphatase from *E*. *coli*) as optimal enzyme candidates [12]. By fine-tuning their expression levels through promoter optimization and gene-order rearrangement, they achieved a pseudouridine titer of 0.511 g L^−1^. Similarly, Zhang et al. expressed *ApsuG* from *Aeromonas hydrophila* and *yjjG* from *E*. *coli* in *E*. *coli* MG1655, reaching a titer of 0.77 g L^−1^ [13]. These studies collectively highlight the importance of enzyme selection and expression balancing for maximizing pseudouridine production.

### 3.2 Promoting pseudouridine production via enhancing intracellular uridine synthesis and inhibiting pseudouridine transport

Aspartate serves as a key precursor in pyrimidine biosynthesis, and its increased availability promotes uracil accumulation [17]. Carbamoyl phosphate synthetase (*carA/B*), which catalyzed carbamoyl phosphate formation from glutamine for pyrimidine synthesis, was negatively regulated by leucyl aminopeptidase (pepA) [18]. Ornithine carbamoyltransferase (argF) diverted carbamoyl phosphate toward citrulline production [19]. In this study, the aspartate kinase gene *thrA* was knocked out to disrupt the metabolic conversion of aspartate to l-aspartate-4-phosphate (Fig. 2D). Thus, genes *pepA* and *argF* were deleted to increase uracil accumulation. Furthermore, the pseudouridine transporter psuT was inactivated to prevent pseudouridine reuptake, and promoting its extracellular accumulation. Building upon *E*. *coli* pRSFDuet-1-*YjjG*-*psuG*, *E*. *coli* Δ*thrA*Δ*psuT*Δ*argF*Δ*pepA* pRSFDuet-1-*YjjG*-*psuG* was constructed through targeted knockout of genes *thrA*, *psuT*, *argF*, and *pepA* (Fig. 2D).

With 5 g L^−1^ uridine supplementation, the engineered strain *E*. *coli* Δ*thrA*Δ*psuT*Δ*argF*Δ*pepA* pRSFDuet-1-*YjjG*-*psuG* achieved 4.76 g L^−1^ pseudouridine at 96 h, representing a 1.29-fold increase over the control strain *E*. *coli* pRSFDuet-1-*YjjG*-*psuG* (3.68 g L^−1^) (Fig. 2E). Notably, compared with the control strain *E*. *coli* pRSFDuet-1-*YjjG*-*psuG*, uridine levels of *E*. *coli* Δ*thrA*Δ*psuT*Δ*argF*Δ*pepA* pRSFDuet-1-*YjjG*-*psuG* remained stable at 0.4 g L^−1^ at 96 h (Fig. 2F), while uracil accumulation increased to 1.55 g L^−1^ (Fig. S3). These results demonstrated that enhanced uracil availability coupled with blocked pseudouridine transport significantly enhanced pseudouridine production in the engineered strain *E*. *coli* Δ*thrA*Δ*psuT*Δ*argF*Δ*pepA* pRSFDuet-1-*YjjG*-*psuG*. Following the knockout of *thrA*, *psuT*, *argF*, and *pepA* genes, a significant adverse effect on cell growth was observed (Fig. S4D). This growth impairment can be primarily attributed to the deletion of *thrA*, which disrupted the biosynthetic flux of key amino acids such as threonine and lysine, thereby inducing metabolic imbalance [20]. Concurrently, knockout of *psuT* was found to strongly inhibit the uptake of extracellular pseudouridine. Although the total pseudouridine production (intracellular and extracellular combined) increased from 3.85 g L^−1^ in the control strain *E*. *coli* pRSFDuet-1-*YjjG*-*psuG* to 5.0 g L^−1^ at 96 h, the proportion of intracellular pseudouridine remained consistently low (<5%) (Fig. S4A), in agreement with previous reports by Zhou et al. [12]. Furthermore, intracellular levels of uridine and uracil were nearly undetectable (Fig. S4B and S4C), suggesting that these metabolites were efficiently channeled into pseudouridine synthesis beyond minimal growth maintenance requirements. Zhou et al. enhanced pseudouridine production in *E*. *coli* MG1655 by disrupting *thrA*, *pepA*, and *argF*, elevating titers from undetectable levels to 0.0259 g L^−1^ [12]. Subsequent overexpression of core pathway enzymes, combined with *psuT* deletion, further increased titer from 0.599 g L^−1^ to 0.642 g L^−1^. In a separate strategy, Wang et al. integrated the *nupC* gene (under control of the P_trc_ promoter) at the gene *yjgX* to improve uridine uptake while deleting *psuT* to block pseudouridine efflux [16]. This dual modification significantly boosted pseudouridine titers from 2.39 g L^−1^ to 3.99 g L^−1^, demonstrating the effectiveness of combined transport engineering and pathway optimization.

### 3.3 Enhancing pseudouridine production via improving uridine catabolism

**Fig. 3:**
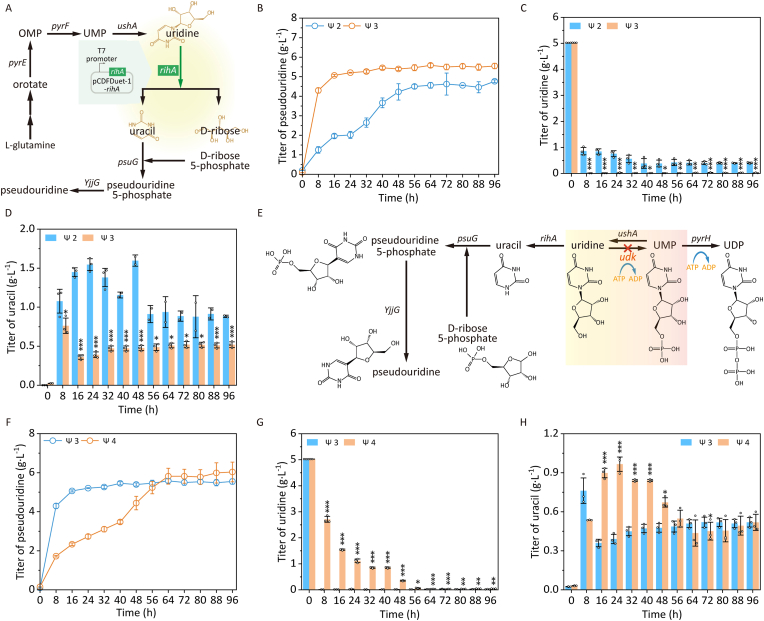
Enhancing pseudouridine production via overexpressing ribonucleoside hydrolase gene *rihA* and knocking out uridine kinase gene *udk*. A, Effect of overexpression of ribonucleoside hydrolase gene *rihA* on pseudouridine production. OMP, orotidine-5P; UMP, Uridine 5′-monophosphate; *pyrE*, orotate phosphoribosyltransferase gene; *pyrF*, orotidine-5′-phosphate decarboxylase gene; *ushA*, 5′-nucleotidase/UDP-sugar diphosphatase gene; *rihA*, pyrimidine-specific ribonucleoside hydrolase gene; *psuG*, pseudouridylate synthase gene; *YjjG*, ΨMP-specific phosphatase gene. B, Titer of pseudouridine. C, Titer of uridine. D, Titer of uracil. Ψ2, *E*. *coli* Δ*thrA*Δ*psuT*Δ*argF*Δ*pepA* pRSFDuet-1-*YjjG*-*psuG*; Ψ3, *E*. *coli* Δ*thrA*Δ*psuT*Δ*argF*Δ*pepA* pRSFDuet-1-*YjjG*-*psuG* pCDFDuet-1-*rihA*. E, Effect of knocking out uridine kinase gene *udk* on pseudouridine production. UMP, Uridine 5′-monophosphate; UDP, Uridine 5′-diphosphate; ATP, Adenosine 5′-triphosphate; ADP, Adenosine 5′-diphosphate; *ushA*, 5′-nucleotidase/UDP-sugar diphosphatase gene; *rihA*, pyrimidine-specific ribonucleoside hydrolase gene; *udk*, uridine kinase gene; *pyrH*, uridylate kinase gene. F, Titer of pseudouridine. G, Titer of uridine. H, Titer of uracil. Ψ3, *E*. *coli* Δ*thrA*Δ*psuT*Δ*argF*Δ*pepA* pRSFDuet-1-*YjjG*-*psuG* pCDFDuet-1-*rihA*; Ψ4, *E*. *coli* Δ*thrA*Δ*psuT*Δ*argF*Δ*pepA*Δ*udk* pRSFDuet-1-*YjjG*-*psuG* pCDFDuet-1-*rihA*. No ∗, *p* > 0.05; ∗, *p* < 0.05; ∗∗, *p* < 0.01; ∗∗∗, *p* < 0.001.

The ribonucleoside hydrolases RihA/B/Cparticipate in uridine catabolism to generate ribose-5-phosphate and uracil in *E*. *coli* [21]. While RihC demonstrates broad substrate specificity for various nucleosides, RihA and RihB exhibit preferential activity toward uridine and cytidine with distinct substrate affinities and RihA was the most efficient hydrolase for uridine conversion to uracil and d-ribose [16,22]. Herein, the recombinant plasmid pCDFDuet-1-*rihA* was constructed to transformed into *E*. *coli* Δ*thrA*Δ*psuT*Δ*argF*Δ*pepA* pRSFDuet-1-*YjjG*-*psuG*, generating the recombinant strain *E*. *coli* Δ*thrA*Δ*psuT*Δ*argF*Δ*pepA* pRSFDuet-1-*YjjG*-*psuG* pCDFDuet-1-*rihA* (Fig. 3A). RihA overexpression significantly enhanced pseudouridine production, with the engineered strain *E*. *coli* Δ*thrA*Δ*psuT*Δ*argF*Δ*pepA* pRSFDuet-1-*YjjG*-*psuG* pCDFDuet-1-*rihA* achieving 5.58 g L^−1^ at 64 h compared to 4.76 g L^−1^ in the control *E*. *coli* Δ*thrA*Δ*psuT*Δ*argF*Δ*pepA* pRSFDuet-1-*YjjG*-*psuG* (1.17-fold increase, Fig. 3B). This improvement coincided with markedly reduced uridine levels, decreasing to near 0 g L^−1^ by 8 h versus 0.86 g L^−1^ in controls (Fig. 3C), confirming efficient uridine hydrolysis. Interestingly, uracil concentrations decreased substantially in the recombinant strain *E*. *coli* Δ*thrA*Δ*psuT*Δ*argF*Δ*pepA* pRSFDuet-1-*YjjG*-*psuG* pCDFDuet-1-*rihA* (0.36 g L^−1^ vs 1.45 g L^−1^ at 16 h, Fig. 3D).

Following overexpression of the *rihA* gene, no significant reduction in cell density was observed during the early cultivation phase (15‒30 h) (Fig. S5). This suggested that high expression of ribonucleoside hydrolase RihA facilitated efficient hydrolysis of uridine, thereby preventing the accumulation of uridine-related growth inhibition and supporting unimpaired cell proliferation. RihA-mediated conversion not only accelerated uridine hydrolysis but also enhanced ribose-5-phosphate generation for pseudouridine biosynthesis while reducing uracil accumulation. In pseudouridine biosynthesis, efficient uridine hydrolysis is critical for redirecting metabolic flux toward the target product. To optimize this step, Wang et al. evaluated three endogenous ribonucleoside hydrolases (RihA, RihB, and RihC), demonstrating that RihA overexpression maximized pseudouridine production (2.39 g L^−1^) [16]. This performance suggested RihA's enhanced catalytic efficiency in uridine cleavage, which amplified uracil and ribose precursor supply and drived higher pseudouridine titers by alleviating substrate limitations. These findings reinforced RihA's pivotal role in pseudouridine pathway engineering.

### 3.4 Enhancing pseudouridine production via knockout of uridine kinase gene udk

The enzymatic phosphorylation of uridine to uridine-5′-monophosphate (UMP), catalyzed by uridine kinase (udk), represented a critical entry point into the pyrimidine salvage pathway [23]. To redirect metabolic flux toward pseudouridine biosynthesis, the uridine kinase gene *udk* was knocked out to construct the engineered strain *E*. *coli* Δ*thrA*Δ*psuT*Δ*argF*Δ*pepA*Δ*udk* pRSFDuet-1-*YjjG*-*psuG* pCDFDuet-1-*rihA* (Fig. 3E). This modification increased pseudouridine production by 8%, reaching 6.04 g L^−1^ at 96 h compared to 5.58 g L^−1^ in the control strain (Fig. 3F). The genetic intervention caused significant uridine accumulation (2.71 g L^−1^ vs 0.01 g L^−1^ at 8 h) due to blocked UMP conversion (Fig. 3G). During early fermentation (16‒48 h), elevated uracil levels were observed in engineered strain *E*. *coli* Δ*thrA*Δ*psuT*Δ*argF*Δ*pepA*Δ*udk* pRSFDuet-1-*YjjG*-*psuG* pCDFDuet-1-*rihA* (Fig. 3H), resulting from enhanced uridine-to-uracil conversion. Meanwhile, between 15 and 40 h of fermentation, a recurrent decline in cell density was observed (Fig. S6). This increased uracil availability subsequently drove higher pseudouridine production through augmented precursor supply. Consistent with Wu et al.‘s findings [17], our study demonstrated that *udk* knockout enhanced uridine accumulation in *E*. *coli*, thereby increasing substrate availability for pseudouridine biosynthesis. This genetic modification effectively redirected metabolic flux toward uridine retention, ultimately boosting pseudouridine production. The concordance between both studies confirmed *udk* deletion as a robust strategy to overcome substrate limitation in nucleoside pathway engineering.

### 3.5 Effect of knocking out uridine phosphorylase gene udp and nucleoside phosphorylase gene ppnp on pseudouridine biosynthesis

**Fig. 4:**
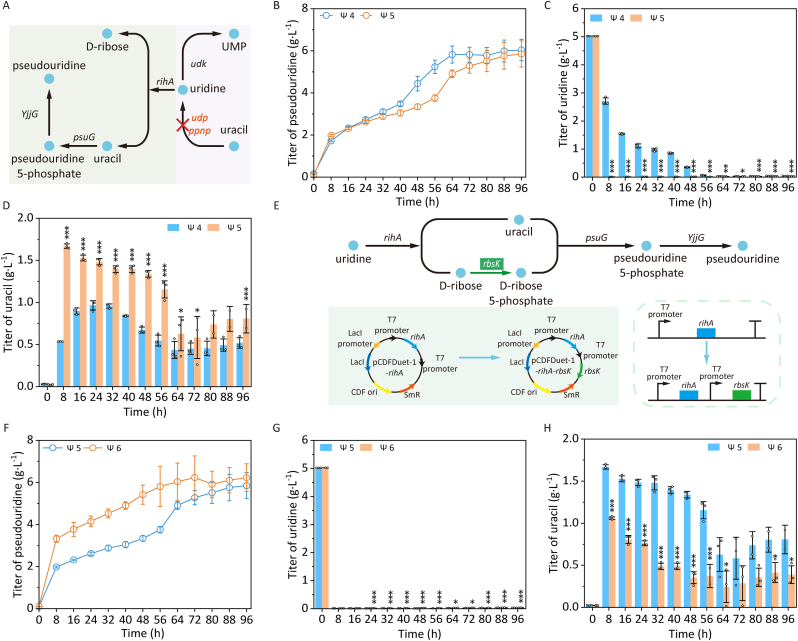
Enhancing pseudouridine production via knocking out genes *udp* and *ppnp*, and overexpressing ribokinase gene *rbsK*. A, Effect of knocking out genes *udp* and *ppnp* on pseudouridine production. UMP, Uridine 5′-monophosphate; *udk*, uridine kinase gene; *udp*, uridine phosphorylase gene; *ppnp*, purine/pyrimidine-nucleoside phosphorylase gene; *rihA*, pyrimidine-specific ribonucleoside hydrolase gene; *psuG*, pseudouridylate synthase gene; *YjjG*, ΨMP-specific phosphatase gene. B, Titer of pseudouridine. C, Titer of uridine. D, Titer of uracil. Ψ4, *E*. *coli* Δ*thrA*Δ*psuT*Δ*argF*Δ*pepA*Δ*udk* pRSFDuet-1-*YjjG*-*psuG* pCDFDuet-1-*rihA*; Ψ5, *E*. *coli* Δ*thrA*Δ*psuT*Δ*argF*Δ*pepA*Δ*udk*Δ*udp*Δ*ppnp* pRSFDuet-1-*YjjG*-*psuG* pCDFDuet-1-*rihA*. E, Increasing pseudouridine production by overexpressing ribokinase gene *rbsK*. *rihA*, pyrimidine-specific ribonucleoside hydrolase gene; *rbsK*, ribokinase gene; *psuG*, pseudouridylate synthase gene; *YjjG*, ΨMP-specific phosphatase gene. F, Titer of pseudouridine. G, Titer of uridine. H, Titer of uracil. Ψ5, *E*. *coli* Δ*thrA*Δ*psuT*Δ*argF*Δ*pepA*Δ*udk*Δ*udp*Δ*ppnp* pRSFDuet-1-*YjjG*-*psuG* pCDFDuet-1-*rihA*; Ψ6, *E*. *coli* Δ*thrA*Δ*psuT*Δ*argF*Δ*pepA*Δ*udk*Δ*udp*Δ*ppnp* pRSFDuet-1-*YjjG*-*psuG* pCDFDuet-1-*rihA-rbsK*. No ∗, *p* > 0.05; ∗, *p* < 0.05; ∗∗, *p* < 0.01; ∗∗∗, *p* < 0.001.

Uridine phosphorylase Udp and nucleoside phosphorylase Ppnp catalyze the cleavage of uridine and other ribonucleosides (e.g., cytidine) to generate corresponding pyrimidine bases and ribose-1-phosphate in *E*. *coli* [24,25], while also facilitating nucleoside interconversion [25]. Herein, the genes *udp* and *ppnp* were knocked out to construct the multi-gene knockout strain *E*. *coli* Δ*thrA*Δ*psuT*Δ*argF*Δ*pepA*Δ*udk*Δ*udp*Δ*ppnp* pRSFDuet-1-*YjjG*-*psuG* pCDFDuet-1-*rihA* (Fig. 4A). The engineering strategy resulted in a pseudouridine titer of 5.85 g L^−1^, representing a slight decrease from 6.04 g L^−1^ observed in the control strain *E*. *coli* Δ*thrA*Δ*psuT*Δ*argF*Δ*pepA*Δ*udk* pRSFDuet-1-*YjjG*-*psuG* pCDFDuet-1-*rihA*. The engineered strain *E*. *coli* Δ*thrA*Δ*psuT*Δ*argF*Δ*pepA*Δ*udk*Δ*udp*Δ*ppnp* pRSFDuet-1-*YjjG*-*psuG* pCDFDuet-1-*rihA* exhibited near-zero uridine concentrations (Fig. 4C), significantly lower than the control, alongside substantially elevated uracil levels (Fig. 4D). Moreover, no adverse effects on cell growth were observed (Fig. S7). These results demonstrated successful blockade of uracil-to-uridine conversion through *udp*/*ppnp* deletion, leading to uracil accumulation. However, the observed pseudouridine reduction suggested potential ribose-5-phosphate limitation, as evidenced by excessive uracil buildup and suboptimal pseudouridine yields, indicating an imbalance in precursor availability for efficient pseudouridine synthesis. Wu et al. demonstrated that *udp* gene knockout significantly enhanced uridine accumulation in their engineered strain [17]. Given that *ppnp* encodes a functional isoenzyme of UDP, *ppnp* was further inactivated to block competing uracil-to-uridine conversion and channel metabolic flux exclusively toward pseudouridine biosynthesis. This dual genetic strategy synergistically maximized precursor availability for pseudouridine production in this work.

### 3.6 Enhance pseudouridine production through overexpression of ribokinase gene rbsK to increase ribose-5-phosphate supply

The biosynthetic of nucleoside analogs critically depends on precursor metabolite availability, with ribose-5-phosphate being a key limiting factor. Ribokinase (RbsK) catalyzes the phosphorylation of d-ribose to ribose-5-phosphate [16], thereby increasing intracellular levels of this essential precursor. To optimize ribose-5-phosphate supply for pseudouridine biosynthetic, the gene *rbsK* was overexpressed in *E*. *coli* Δ*thrA*Δ*psuT*Δ*argF*Δ*pepA*Δ*udk*Δ*udp*Δ*ppnp* pRSFDuet-1-*YjjG*-*psuG* pCDFDuet-1-*rihA*, generating strain *E*. *coli* Δ*thrA*Δ*psuT*Δ*argF*Δ*pepA*Δ*udk*Δ*udp*Δ*ppnp* pRSFDuet-1-*YjjG*-*psuG* pCDFDuet-1-*rihA-rbsK* (Fig. 4E). This modification increased pseudouridine production from 5.85 g L^−1^ to 6.23 g L^−1^ (Fig. 4F), demonstrating enhanced ribose-5-phosphate synthesis that further promoted pseudouridine production. Notably, uridine levels remained consistently low (0.01 g L^−1^ at 8 h, Fig. 4G), confirming that overexpressing the *rbsK* gene promoted d-ribose consumption without uridine accumulation. Furthermore, in comparison to the control strain, the engineered strain *E*. *coli* Δ*thrA*Δ*psuT*Δ*argF*Δ*pepA*Δ*udk*Δ*udp*Δ*ppnp* pRSFDuet-1-*YjjG*-*psuG* pCDFDuet-1-*rihA-rbsK* demonstrated a significant reduction in uracil accumulation, reaching a maximum level at 1.06 g L^−1^ at 8 h (Fig. 4H). These results indicated that overexpression of the *rbsK* gene not only enhanced the efficient biosynthesis of the pseudouridine precursor ribose-5-phosphate but also accelerated the metabolic utilization of another precursor uracil, consequently enhancing the overall production efficiency of pseudouridine. Meanwhile, YjjG, PsuG, RihA, and RbsK have been successfully expressed, and the growth of recombinant *E*. *coli* was not significantly inhibited (Fig. S8 and S9). Moreover, previous studies have also validated the critical role of RbsK in enhancing pseudouridine biosynthesis. Wang et al. found that coordinated overexpression of YeiN (pseudouridine-5′-phosphate glycosylase), RbsK (ribokinase), and RihB (ribonucleoside hydrolase) in *E*. *coli* achieved a pseudouridine titer of 1.93 g L^−1^ [16]. These findings confirmed that RbsK-mediated phosphorylation of ribose significantly contributed to pseudouridine production by improving the availability of phosphorylated sugar precursors for nucleotide biosynthesis.

### 3.7 Increasing pseudouridine production using two-stage uridine supplementation strategy

**Fig. 5:**
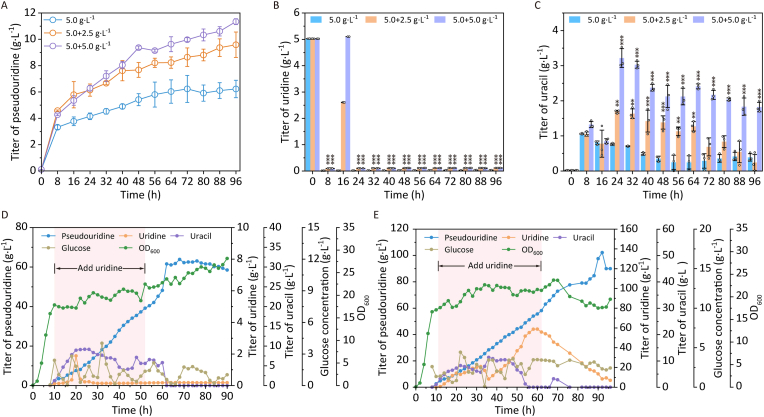
Enhancing pseudouridine production via two-stage uridine supplementation strategy and efficient production of pseudouridine by optimized fed-batch strategy in a 5-L bioreactor. A, Titer of pseudouridine in shaking flasks. B, Titer of uridine in shaking flasks. C, Titer of uracil in shaking flasks. D, Supplementing 70 g L^−1^ uridine (final concentration) in a 5-L bioreactor. E, Supplementing 120 g L^−1^ uridine (final concentration) in a 5-L bioreactor. No ∗, *p* > 0.05; ∗∗, *p* < 0.01; ∗∗∗, *p* < 0.001.

To further enhance pseudouridine production in recombinant strain *E*. *coli* Δ*thrA*Δ*psuT*Δ*argF*Δ*pepA*Δ*udk*Δ*udp*Δ*ppnp* pRSFDuet-1-*YjjG*-*psuG* pCDFDuet-1-*rihA-rbsK*, a two-stage uridine supplementation strategy was implemented. The tested supplementation regiments were: (i) 5.0 g L^−1^ (at 0 h) + 2.5 g L^−1^ (at 16 h) and (ii) 5.0 g L^−1^ (at 0 h) + 5.0 g L^−1^ (at 16 h). Under the first supplementation strategy (5.0 + 2.5 g L^−1^), pseudouridine titer was increased from the control level of 6.23 g L^−1^ to 9.59 g L^−1^ (Fig. 5A). When the supplementation was increased to 5.0 + 5.0 g L^−1^, the titer further increased to 11.34 g L^−1^, representing a 1.82-fold enhancement compared to the control. As shown in Fig. 5B, the recombinant strain *E*. *coli* Δ*thrA*Δ*psuT*Δ*argF*Δ*pepA*Δ*udk*Δ*udp*Δ*ppnp* pRSFDuet-1-*YjjG*-*psuG* pCDFDuet-1-*rihA-rbsK* efficiently metabolized uridine, maintaining its concentration at very low levels (<0.15 g L^−1^) under both supplementation strategies. However, uracil accumulation increased significantly, particularly with the strategy (5.0 + 5.0 g L^−1^), reaching 1.82 g L^−1^ at 96 h (Fig. 5C). Notably, excessive uridine supplementation did not suppress the growth of the recombinant strain *E*. *coli* Δ*thrA*Δ*psuT*Δ*argF*Δ*pepA*Δ*udk*Δ*udp*Δ*ppnp* pRSFDuet-1-*YjjG*-*psuG* pCDFDuet-1-*rihA-rbsK* (Fig. S8). During early fermentation stage (<70 h), OD_600_ of the supplemented cultures was lower than that of the control, likely due to transient growth inhibition by high uridine levels. However, in later fermentation stages (>70 h), OD_600_ of recombinant strain *E*. *coli* Δ*thrA*Δ*psuT*Δ*argF*Δ*pepA*Δ*udk*Δ*udp*Δ*ppnp* pRSFDuet-1-*YjjG*-*psuG* pCDFDuet-1-*rihA-rbsK* surpassed the control, suggesting that the cells metabolized the excess uridine, alleviating inhibition and enabling further biomass accumulation.

### 3.8 Enhanced pseudouridine production via optimized fed-batch strategy in a 5-L bioreactor

The recombinant strain *E*. *coli* Δ*thrA*Δ*psuT*Δ*argF*Δ*pepA*Δ*udk*Δ*udp*Δ*ppnp* pRSFDuet-1-*YjjG*-*psuG* pCDFDuet-1-*rihA-rbsK* demonstrated high-efficiency pseudouridine production in a 5-L bioreactor under optimized fed-batch strategy. As shown in Fig. 5D, the fermentation temperature was lowered to 25 °C at 8 h, followed by co-supplementation of IPTG and uridine (from 8 h to 54 h). At a final uridine concentration of 70 g L^−1^, the maximum pseudouridine titer reached 63.9 g L^−1^ at 68 h, achieving a uridine conversion rate of 91.3%. When the supplemented uridine concentration was increased to 120 g L^−1^, pseudouridine titer peaked at 102.2 g L^−1^ by 92 h (Fig. 5E), which was the highest reported level to date (Table S5). Zhang et al. constructed an engineered *E*. *coli* MG1655 strain by deleting *pgi* and *psuT*, overexpressing *ApsuG*, *yjjG*, *zwf*, *UraA*, and *PelB*, and optimizing the RBS sequence, achieving a pseudouridine titer of 27.5 g L^−1^ in a 5-L bioreactor via fed-batch uracil and glucose supplementation [13]. Yu et al. engineered *E*. *coli* MB219 by deleting seven genes (*pbs1*, *psuK*, *nupC*, *nupG*, *psuT*, *rutA*, and *upp*) and overexpressing five genes (*NmYgdH*, *RjPsuG*, *HDHD1*, *zwf*, and *gnd*) via an antibiotic-free plasmid system, achieving a pseudouridine titer of 45.3 g L^−1^ in a 5-L bioreactor with fed-batch glucose [26]. Notably, the conversion rate remained consistently high (91.6%) in this study, demonstrating that elevated uridine conversions did not impair that strain's conversion efficiency, maintaining a 90% conversion rate comparable to supplementation of 70 g L^−1^ uridine. The marked accumulation of uridine observed between 50 h and 70 h in the 5-L bioreactor is likely attributable to an excessive feeding rate during that period.

As shown in Fig. S8A and S8C, under both uridine supplementation strategies, the specific growth rate increased sharply and reached a peak during the early fermentation phase (0‒10 h), followed by a rapid decline and stabilization at a markedly low level. This transition coincided with the timing of temperature reduction and the addition of both IPTG and uridine. The observed growth retardation was likely attributable to a combination of factors, including decreased temperature, IPTG toxicity, and metabolic stress induced by high uridine concentration. The specific pseudouridine production rate differed significantly between the two conditions (Fig. S10B and S10D). With supplementation of 70 g L^−1^ uridine, although a transient peak emerged around 40 h, the overall specific growth rate remained near zero. Correspondingly, pseudouridine accumulation increased almost linearly with the fermentation time (Fig. S10D). In contrast, under supplementation with 120 g L^−1^ uridine, a brief acceleration in early-stage growth correlated with a sharp rise in the specific production rate. However, as severe growth inhibition set in during later stages, the specific production rate declined and eventually approached zero. These results confirmed that high uridine supplementation did not significantly hinder the strain's capacity for efficient pseudouridine biosynthesis.

## 4 Conclusions

This study successfully constructed an engineered *E*. *coli* strain capable of high-yield pseudouridine production through systematic metabolic engineering. Key strategies included overexpression of the core biosynthetic enzyme genes (*psuG* and *YjjG*), targeted knockout of competing pathway and transporter genes (*thrA*, *psuT*, *argF*, *pepA*, *udk*, *udp*, *ppnp*) to redirect metabolic flux, and enhanced precursor supply via overexpression of *rihA* and *rbsK*. The principal achievements are as follows: (i) Establishment and reinforcement of the psuG/YjjG-based pseudouridine synthesis pathway, with uridine supplementation significantly boosting production; (ii) Increased uracil accumulation through knockout of *thrA*, *argF*, and *pepA*, coupled with blocking product reuptake via *psuT* deletion to promote extracellular pseudouridine accumulation; (iii) Enhanced uridine hydrolysis through *rihA* overexpression and prevention of uridine salvage phosphorylation via *udk* knockout, synergistically directing metabolic flux toward the target product; (iv) Alleviation of ribose-5-phosphate limitation by overexpressing *rbsK*; (v) A two-stage uridine feeding strategy in shake flasks increasing the titer to 11.34 g L^−1^; (vi) Optimized fed-batch fermentation in a 5-L bioreactor achieving a record-high pseudouridine titer of 102.2 g L^−1^ with a conversion efficiency exceeding 91%. In summary, this work integrates innovations across pathway construction, metabolic regulation, precursor supply, and process optimization, establishing an efficient cell factory for pseudouridine biosynthesis and providing a critical technological foundation for its industrial-scale biomanufacturing. However, multiple gene deletions and plasmid-based overexpression can impose a significant metabolic burden on the host. This often compromises plasmid stability and creates a growth-production trade-off, where resources diverted for recombinant protein synthesis reduce biomass formation and overall productivity. Chromosomal integration offers a promising alternative by eliminating selective pressure, enhancing genetic stability, and alleviating metabolic load, thereby supporting more robust and scalable strain performance. In our future work, chromosomal integration will be employed to construct engineered *E*. *coli* strains for the efficient biosynthesis of pseudouridine. Meanwhile, pseudouridine can be efficiently recovered from fermentation broth using established techniques such as activated carbon adsorption, ion-exchange chromatography, and crystallization. Its favorable physicochemical properties—including high water solubility and stability under acidic and thermal conditions—support scalable recovery. Crystallization, in particular, offers a cost-effective route to achieve the purity required for pharmaceutical and mRNA applications, underscoring the translational potential of our platform. In parallel, we are currently employing this method to purify pseudouridine from fermentation broth and obtain it in crystalline form.

## CRediT authorship contribution statement

**Jing Song:** Writing – original draft, Investigation. **Wei Shen:** Writing – review & editing. **Yuanyuan Xia:** Writing – review & editing. **Li Zhou:** Writing – review & editing. **Yingjun Zhou:** Writing – review & editing, Methodology. **Haiquan Yang:** Writing – review & editing, Writing – original draft, Methodology. **Xianzhong Chen:** Writing – review & editing, Methodology.

## Funding

This work was funded by the Key Research and Development Program of China (2024YFA0918300), and the 10.13039/501100013314111 Project (111-2-06).

## Declaration of competing interest

The authors declare that they have no known competing financial interests or personal relationships that could have appeared to influence the work reported in this paper.
