# Supplementary material for: Systematic metabolic engineering of Escherichia coli for high-level production of pseudouridine via pathway optimization and precursor enhancement
Source: Synth Syst Biotechnol. 2026 Apr 6;14:32–40. doi: 10.1016/j.synbio.2026.03.013 (PMC13090712; doi:10.1016/j.synbio.2026.03.013)
Supplement: Multimedia component 1 [file mmc1.docx]

**Systematic metabolic engineering of *Escherichia coli* for high-level production of pseudouridine via pathway optimization and precursor enhancement**

Jing Song^1^, Wei Shen^2^, Yuanyuan Xia^2^, Li Zhou^2^, Yingjun Zhou^3^, Haiquan Yang^1,*^, Xianzhong Chen^2,*^

^1^ School of Biotechnology and Key Laboratory of Carbohydrate Chemistry and Biotechnology of Ministry of Education, Jiangnan University, Wuxi 214122, China

^2^ School of Biotechnology and Key Laboratory of Industrial Biotechnology of Ministry of Education, Jiangnan University, Wuxi 214122, China

^3^ Xiangya School of Pharmaceutical Sciences, Central South University, Changsha 410013, China

*Corresponding author. E-mail: haiquanyang@jiangnan.edu.cn (H.Y.); xzchen@jiangnan.edu.cn (X.C.)

Table S1 Strains used and constructed in this study

| Strains | Characteristics | Sources |
| --- | --- | --- |
| *E*. *coli* BL21 | Wild-type *E*. *coli* BL21(DE3) | Novagen |
| *E*. *coli* pRSFDuet-1-*YjjG*-*psuG* | *E*. *coli* BL21(DE3) derivative, containing the plasmid pRSFDuet-1-*YjjG*-*psuG* | This study |
| *E*. *coli* Δ*thrA*Δ*psuT*Δ*argF*Δ*pepA* pRSFDuet-1-*YjjG*-*psuG* | *E*. *coli* pRSFDuet-1-*YjjG*-*psuG* derivative, deleting the genes *thrA*, *psuT*, *argF*, and *pepA* | This study |
| *E*. *coli* Δ*thrA*Δ*psuT*Δ*argF*Δ*pepA* pRSFDuet-1-*YjjG*-*psuG* pCDFDuet-1-*rihA* | *E*. *coli* Δ*thrA*Δ*psuT*Δ*argF*Δ*pepA* pRSFDuet-1-*YjjG*-*psuG* derivative, containing the plasmid pCDFDuet-1-*rihA* | This study |
| *E*. *coli* Δ*thrA*Δ*psuT*Δ*argF*Δ*pepA*Δ*udk* pRSFDuet-1-*YjjG*-*psuG* pCDFDuet-1-*rihA* | *E*. *coli*Δ*thrA*Δ*psuT*Δ*argF*Δ*pepA* pRSFDuet-1-*YjjG*-*psuG* pCDFDuet-1-*rihA* derivative, deleting the gene *udk* | This study |
| *E*. *coli* Δ*thrA*Δ*psuT*Δ*argF*Δ*pepA*Δ*udk*Δ*udp*Δ*ppnp* pRSFDuet-1-*YjjG*-*psuG* pCDFDuet-1-*rihA* | *E*. *coli* Δ*thrA*Δ*psuT*Δ*argF*Δ*pepA*Δ*udk* pRSFDuet-1-*YjjG*-*psuG* pCDFDuet-1-*rihA* derivative, deleting the genes *udp* and *ppnp* | This study |
| *E*. *coli* Δ*thrA*Δ*psuT*Δ*argF*Δ*pepA*Δ*udk*Δ*udp*Δ*ppnp* pRSFDuet-1-*YjjG*-*psuG* pCDFDuet-1-*rihA-rbsK* | *E*. *coli* Δ*thrA*Δ*psuT*Δ*argF*Δ*pepA*Δ*udk*Δ*udp*Δ*ppnp* pRSFDuet-1-*YjjG*-*psuG* derivative, containing the plasmid pCDFDuet-1-*rihA-rbsK* | This study |
| *E*. *coli* pTargetF-*thrA* | *E*. *coli* BL21 derivative, containing the plasmid pTargetF-*thrA* | This study |
| *E*. *coli* pTargetF-*psuT* | *E*. *coli* BL21 derivative, containing the plasmid pTargetF-*psuT* | This study |
| *E*. *coli* pTargetF-*argF* | *E*. *coli* BL21 derivative, containing the plasmid pTargetF-*argF* | This study |
| *E*. *coli* pTargetF-*pepA* | *E*. *coli* BL21 derivative, containing the plasmid pTargetF-*pepA* | This study |
| *E*. *coli* pTargetF-*udk* | *E*. *coli* BL21 derivative, containing the plasmid pTargetF-*udk* | This study |
| *E*. *coli* pTargetF-*udp* | *E*. *coli* BL21 derivative, containing the plasmid pTargetF-*udp* | This study |
| *E*. *coli* pTargetF-*ppnp* | *E*. *coli* BL21 derivative, containing the plasmid pTargetF-*ppnp* | This study |
| *E*. *coli* pCas | *E*. *coli* BL21 derivative, containing the plasmid pCas | This study |

Table S2 Plasmids constructed and used in this study

| Plasmids | Characteristics | Sources |
| --- | --- | --- |
| pRSFDuet-1 | Expression plasmid, T7 promoter, Kan^R^ | Novagen |
| pRSFDuet-1-*YjjG* | Plasmid pRSFDuet-1 derivative, including the gene *YjjG* | This study |
| pRSFDuet-1-*YjjG*-*psuG* | Plasmid pRSFDuet-1 derivative, including the genes *YjjG* and *psuG* | This study |
| pCDFDuet-1 | Expression plasmid, T7 promoter, Sm^R^ | Novagen |
| pCDFDuet-1-*rihA* | Plasmid pCDFDuet-1 derivative, including the gene *rihA* | This study |
| pCDFDuet-1-*rihA-rbsK* | Plasmid pCDFDuet-1 derivative, including the genes *rihA* and *rbsK* | This study |
| pTargetF | pMB1 aadA sgRNA, spe^R^ | Novagen |
| pTargetF-*thrA* | pTargetF derivative, including the sgRNA-*thrA* | This study |
| pTargetF-*psuT* | pTargetF derivative, including the sgRNA-*psuT* | This study |
| pTargetF-*argF* | pTargetF derivative, including the sgRNA-*argF* | This study |
| pTargetF-*pepA* | pTargetF derivative, including the sgRNA-*pepA* | This study |
| pTargetF-*udk* | pTargetF derivative, including the sgRNA-*udk* | This study |
| pTargetF-*udp* | pTargetF derivative, including the sgRNA-*udp* | This study |
| pTargetF-*ppnp* | pTargetF derivative, including the sgRNA-*ppnp* | This study |
| pCas | repA101(Ts) kan Pcas-cas9 ParaB-Red lacI^q^-P_trc_-sgRNA-pMB1 | Novagen |

Table S3 Primers used in this study

| Primers | Sequences (5’→3’) | Restriction endonuclease sites |
| --- | --- | --- |
| *YjjG*-FW | GAATTCAATGAAGTGGGACTGGATTTTC | *Eco*R I |
| *YjjG*-RS | AAGCTTTCAGTGTTTACACAGGAGC | *Hind* III |
| *psuG*-FW | GATATCAATGTCTGAATTAAAAATTTCCC | *Eco*R V |
| psuG-RS | GGTACCTTAACCCGCGAGACGCTGA | *Kpn* I |
| *rihA*-FW | GAATTCAATGGCACTGCCAATTCTGTT | *Eco*R I |
| *rihA*-RS | AAGCTTTTAAGCGTAAAATTTCAGACGAT | *Hind* III |
| *rbsK*-FW | GATATCGATGCAAAACGCAGGCAGCC | *Eco*R V |
| *rbsK*-RS | GGTACCTCACCTCTGCCTGTCTAAAAA | *Kpn* I |
| Δ*thrA-*up-FW | AGCTTTTCATTCTGACTGCA |  |
| Δ*thrA-*up-RS | GGGCATAAACTTTAACCATGGGTTGTTACCTCGTTACCTT |  |
| Δ*thrA-*down-FW | AAGGTAACGAGGTAACAACCCATGGTTAAAGTTTATGCCC |  |
| Δ*thrA-*down-RS | CCACTCATCAAACCCTGGC |  |
| *sg-thrA*-FW | GTCCTAGGTATAATACTAGTCTGGAAAGCAATGCCAGGCAGTTTTAGAGCTAGAAATAGC |  |
| *sg-thrA*-RS | ACTAGTATTATACCTAGGACTGAGC |  |
| Δ*thrA*-RCR-FW | AGCTTTTCATTCTGACTGCA |  |
| Δ*thrA*-RCR-RS | GATAACATCTTTCATCAGCTT |  |
| Δ*psuT-*up-FW | ATGGATATAATGAGAAGTGTTG |  |
| Δ*psuT-*up-RS | CCGCCGATAATGCCGTTGATTCTGGAAAATGCTGCCAAG |  |
| Δ*psuT-*down-FW | CTTGGCAGCATTTTCCAGAATCAACGGCATTATCGGCGG |  |
| Δ*psuT-*down-RS | TTACGCCAGACCAATAAAGA |  |
| *sg-psuT*-FW | GTCCTAGGTATAATACTAGTCATCATGCTCTACTTCCCACGTTTTAGAGCTAGAAATAGC |  |
| *sg-psuT*-RS | ACTAGTATTATACCTAGGACTGAGC |  |
| Δ*psuT*-RCR-FW | TAGAAACCTTCGGTGTGCC |  |
| Δ*psuT*-RCR-RS | GATTGAATCTTACCAACCGC |  |
| Δ*argF-*up-FW | AAGTAAGTGCCCCAACCGTC |  |
| Δ*argF-*up-RS | GGCATCTTTATAGCGATATCAAAGATCCCTCCTGTGGCTAAC |  |
| Δ*argF-*down-FW | GTTAGCCACAGGAGGGATCTTTGATATCGCTATAAAGATGCC |  |
| Δ*argF-*down-RS | GATTTTAACTTAAGCAATCAAGAAA |  |
| *sg-argF*-FW | GTCCTAGGTATAATACTAGTCATACATGCGACCCAGCACGGTTTTAGAGCTAGAAATAGC |  |
| *sg-argF*-RS | ACTAGTATTATACCTAGGACTGAGC |  |
| Δ*argF*-RCR-FW | AAGTAAGTGCCCCAACCGTC |  |
| Δ*argF*-RCR-RS | GTATGCATGCTCCGTGATAAAT |  |
| Δ*pepA-*up-FW | ACAGTTTGCCCAGCGTCAT |  |
| Δ*pepA-*up-RS | CAGCCTTGCCTGACGCAAGCACTACGCTCCTGAATCT |  |
| Δ*pepA-*down-FW | AGATTCAGGAGCGTAGTGCTTGCGTCAGGCAAGGCTG |  |
| Δ*pepA-*down-RS | AGCCTGCTTTTCATCTTCAC |  |
| *sg-pepA*-FW | GTCCTAGGTATAATACTAGTGAGATGAAGTACGATATGTGGTTTTAGAGCTAGAAATAGC |  |
| *sg-pepA*-RS | ACTAGTATTATACCTAGGACTGAGC |  |
| Δ*pepA*-RCR-FW | ACAGTTTGCCCAGCGTCAT |  |
| Δ*pepA*-RCR-RS | TAATCAATATATCGCGCGGG |  |
| Δ*udk-*up-FW | GTAGAACTCCAGCACAATGC |  |
| Δ*udk-*up-RS | GGTTAATCAGGTCGCTAAATATGCTTGATAAATTGTGTACCGTT |  |
| Δ*udk-*down-FW | AACGGTACACAATTTATCAAGCATATTTAGCGACCTGATTAACC |  |
| Δ*udk-*down-RS | GGCATGGCGATAAAACGCC |  |
| *sg-udk*-FW | GTCCTAGGTATAATACTAGTCGTGAATTGCGTGAGCAAGTGTTTTAGAGCTAGAAATAGC |  |
| *sg-udk*-RS | ACTAGTATTATACCTAGGACTGAGC |  |
| Δ*udk*-RCR-FW | TATCCTCAATGGGCCTGAATT |  |
| Δ*udk*-RCR-RS | GGCATGGCGATAAAACGCC |  |
| Δ*udp-*up-FW | TACCCCTCCAGCGCCAGA |  |
| Δ*udp-*up-RS | CGAAGTTGGGGAGCGTTTTATACAACTCCTCTGTGAATCG |  |
| Δ*udp-*down-FW | CGATTCACAGAGGAGTTGTATAAAACGCTCCCCAACTTCG |  |
| Δ*udp-*down-RS | ATGCAACCAGGCCCGCTG |  |
| *sg-udp*-FW | GTCCTAGGTATAATACTAGTCTCACTAAAAACGATTTACAGTTTTAGAGCTAGAAATAGC |  |
| *sg-udp*-RS | ACTAGTATTATACCTAGGACTGAGC |  |
| Δ*udp*-RCR-FW | ATACAGCCAGGTGATACGTC |  |
| Δ*udp*-RCR-RS | ATGCAACCAGGCCCGCTG |  |
| Δ*ppnp-*up-FW | ACCTGCAAGGTGTGGTTGA |  |
| Δ*ppnp-*up-RS | TCAAGGGGAAGGCGAGGAAAAACTGGCCCTGTCTGGTA |  |
| Δ*ppnp-*down-FW | TACCAGACAGGGCCAGTTTTTCCTCGCCTTCCCCTTGA |  |
| Δ*ppnp-*down-RS | GCGCAGCAATATTATGCGTT |  |
| *sg-ppnp*-FW | GTCCTAGGTATAATACTAGTTCCGGCAAAGTGAAATCAATGTTTTAGAGCTAGAAATAGC |  |
| *sg-ppnp*-RS | ACTAGTATTATACCTAGGACTGAGC |  |
| Δ*ppnp*-RCR-FW | TTCCCATCATAGCCTGCTG |  |
| Δ*ppnp*-RCR-RS | GCGCAGCAATATTATGCGTT |  |

Table S4 Gene ID of key genes

| Genes | Gene ID |
| --- | --- |
| *YjjG* | 948899 |
| *psuG* | 946699 |
| *rihA* | 945503 |
| *rbsK* | 948260 |

Table S5 Comparison of pseudouridine titers achieved in this study with those reported for fermentation-based production

| Initial strains | Key modifications | Fermentation conditions | Titer (g·L^-1^) | References |  |
| --- | --- | --- | --- | --- | --- |
| *E*. *coli* BL21 (DE3) | Knocking out genes *thrA*, *psuT*, *argF*, *pepA*, *udk*, *udp*, and *ppnp*; Overexpressing genes *psuG*, *YjjG*, *rihA*, and *rbsK* using plasmids | Fed-batch fermentation in a 5-L bioreactor using fermentation medium with 4 mL·L^-1^ glycerol, with temperature shifted to 25°C and 0.8 mM IPTG added at 8 h, followed by uridine feeding to 120 g·L^-1^ under maintained conditions of pH 7.0, 30‒50% DO, and glucose fed in batches to keep levels below 3 g·L^-1^. | 102.2 | This study |  |
| *E*. *coli* MG1655 | Knocking out genes *argF*, *thrA*, *pepA*, and *psuT*; Overexpressing genes *RspsuG* and *yjjG* | Fed-batch fermentation in a 5-L bioreactor using fermentation medium with 10 g·L^-1^ glucose. At 8 h, temperature was shifted to 30°C and 0.1 mM IPTG added, with pH maintained at 7.0, DO > 25%, and glucose fed in batches to keep levels below 5 g·L^-1^. | 7.9 | [12] |  |
| *E*. *coli* MG1655 | Knocking out genes *pgi* and *psuT*; Overexpressing genes *ApsuG*, *yjjG*, *zwf*, *UraA*, and *PelB*; Optimizing RBS sequence | In a 5-L fed-batch bioreactor with initial glycerol at 10 g·L^-1^, temperature was shifted to 20°C and 0.2 mM IPTG added at OD_600_ 14–16; thereafter, glucose was held at 10 g·L^-1^ and uracil fed in batches to keep < 5 g·L^-1^. | 27.5 | [13] |  |
| *E*. *coli* MB219 (UMP-high producing derivative strain) | Knocking out genes *pbs1*, *psuK*, *nupC*, *nupG*, *psuT*, *rutA*, and *upp*; Overexpressing genes *NmYgdH*, *RjPsuG*, *HDHD1*, *zwf*, and *gnd* via an antibiotic-free screening plasmid expression system | Fed-batch fermentation in a 5-L bioreactor with initial glucose at 20 g·L^-1^, maintained at 37°C, pH 7.0, and 30% DO, with glucose fed intermittently to sustain carbon supply. | 45.3 | [26] |  |


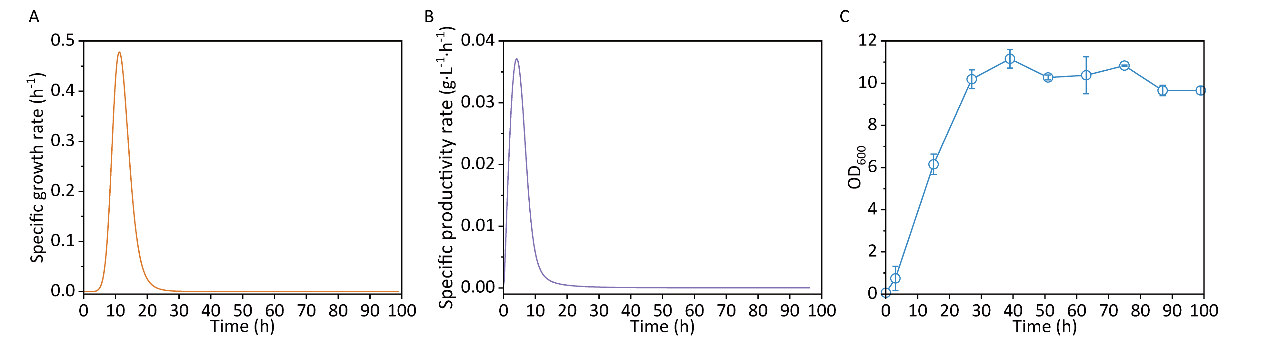


**Figure S1** Specific growth rate, specific productivity rate, and cell density of *E*. *coli* pRSFDuet-1-*YjjG*-*psuG* during *de novo* synthesis of pseudouridine (without uridine supplementation). A, Specific growth rate. B, Specific production rate. C, Growth curve.


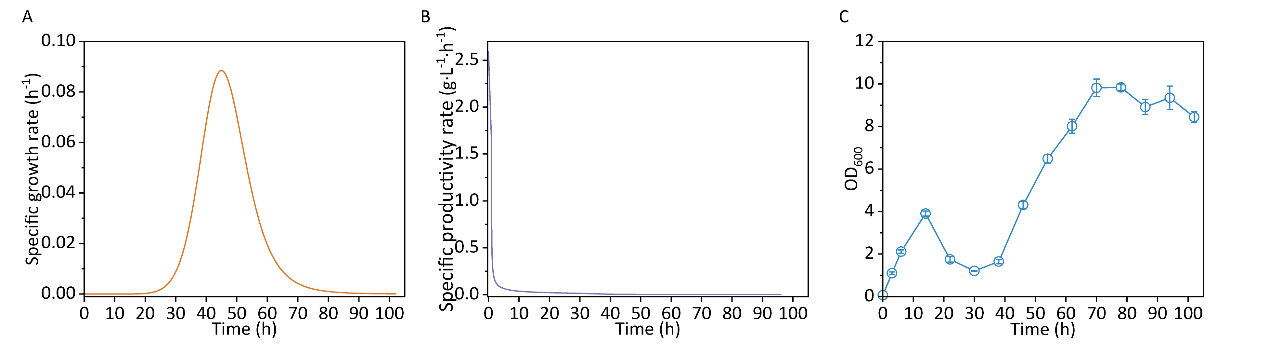


**Figure** **S2** Specific growth rate, specific productivity rate, and cell density of *E*. *coli* pRSFDuet-1-*YjjG*-*psuG* during pseudouridine synthesis with 5 g·L^-1^ uridine supplementation. A, Specific growth rate. B, Specific production rate. C, Growth curve.


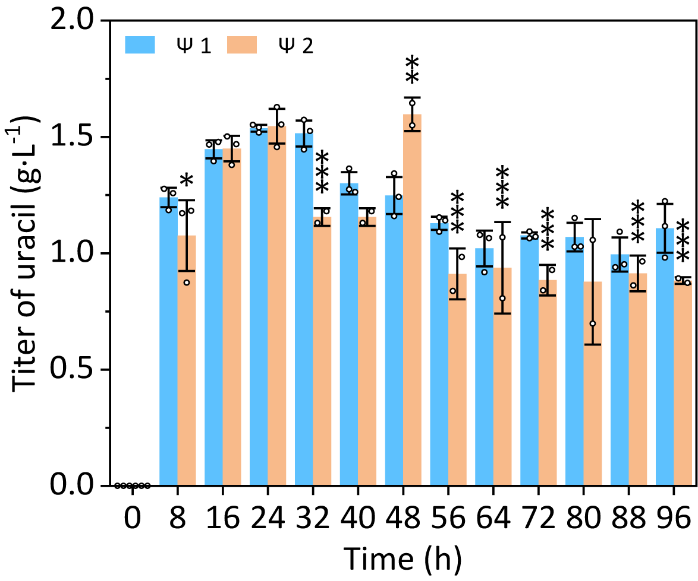


**Figure S3** Effect of enhancing extracellular uridine biosynthesis and inhibiting pseudouridine transport on uracil accumulation. Ψ1, *E*. *coli* pRSFDuet-1-*YjjG*-*psuG*; Ψ2, *E*. *coli* Δ*thrA*Δ*psuT*Δ*argF*Δ*pepA* pRSFDuet-1-*YjjG*-*psuG*. No *, *p* > 0.05; *, *p* < 0.05; **, *p* < 0.01; ***, *p* < 0.001.


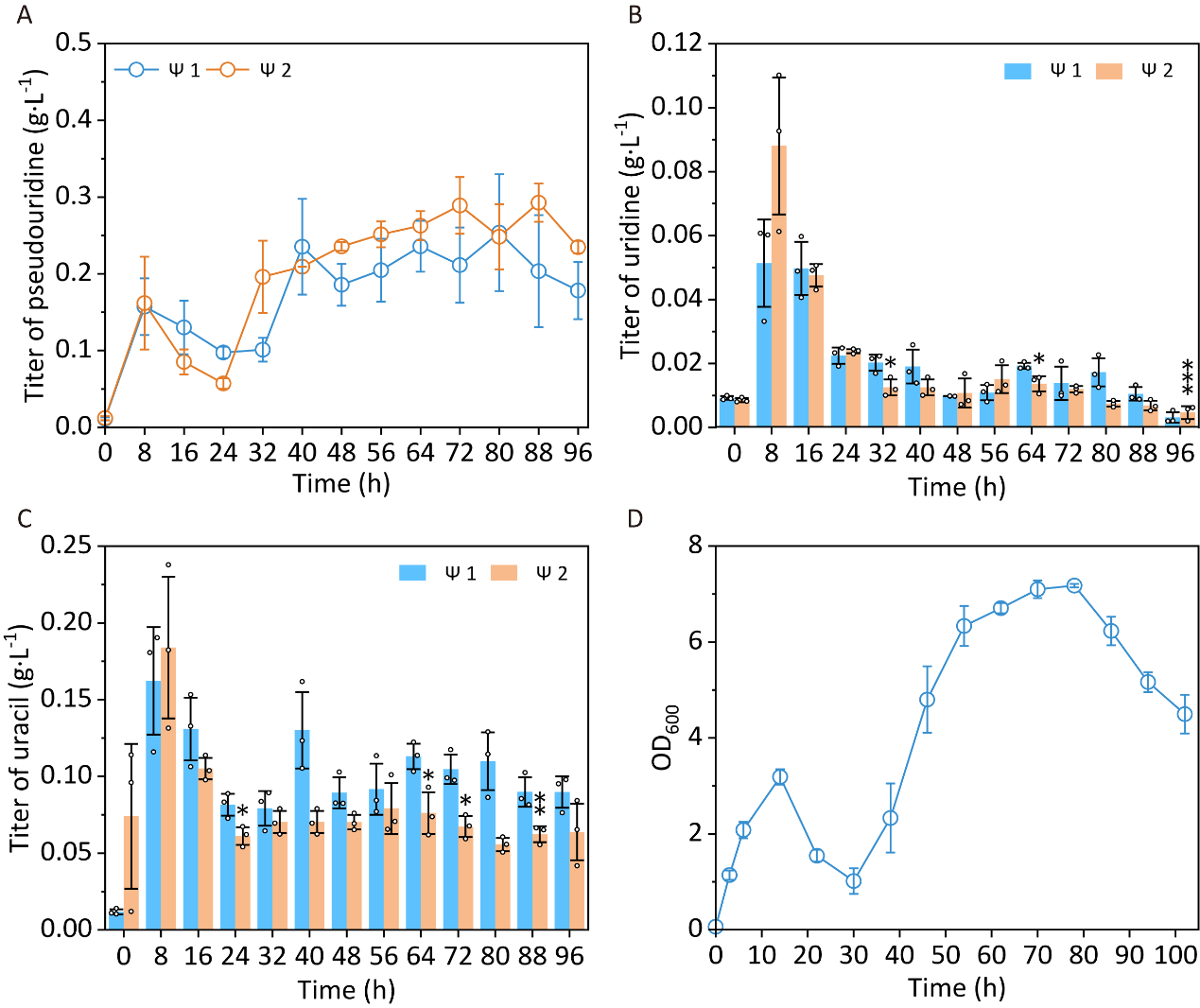


**Figure S4** Effect of enhancing intracellular uridine biosynthesis and inhibiting pseudouridine transport on pseudouridine production, uridine accumulation, uracil accumulation, and cell density. A, Titer of pseudouridine. B, Titer of uridine. C, Titer of uracil. D, Growth curve of *E*. *coli* Δ*thrA*Δ*psuT*Δ*argF*Δ*pepA* pRSFDuet-1-*YjjG*-*psuG*. Ψ1, *E*. *coli* pRSFDuet-1-*YjjG*-*psuG*; Ψ2, *E*. *coli* Δ*thrA*Δ*psuT*Δ*argF*Δ*pepA* pRSFDuet-1-*YjjG*-*psuG*. No *, *p* > 0.05; *, *p* < 0.05; **, *p* < 0.01; ***, *p* < 0.001.


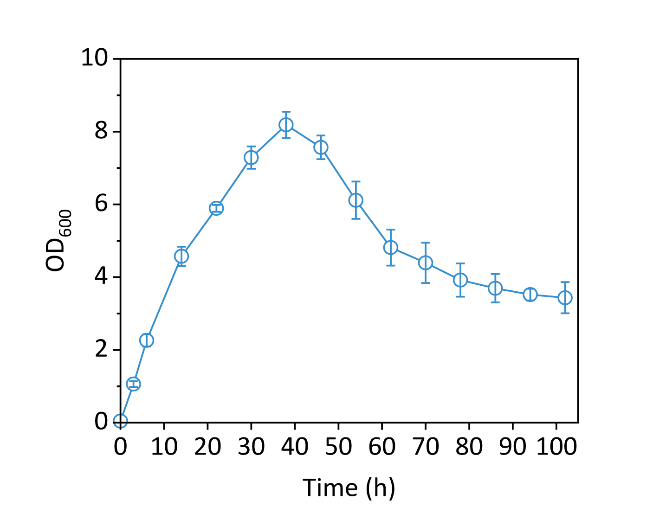


**Figure S5** Growth curve of *E*. *coli* Δ*thrA*Δ*psuT*Δ*argF*Δ*pepA* pRSFDuet-1-*YjjG*-*psuG* pCDFDuet-1-*rihA*.


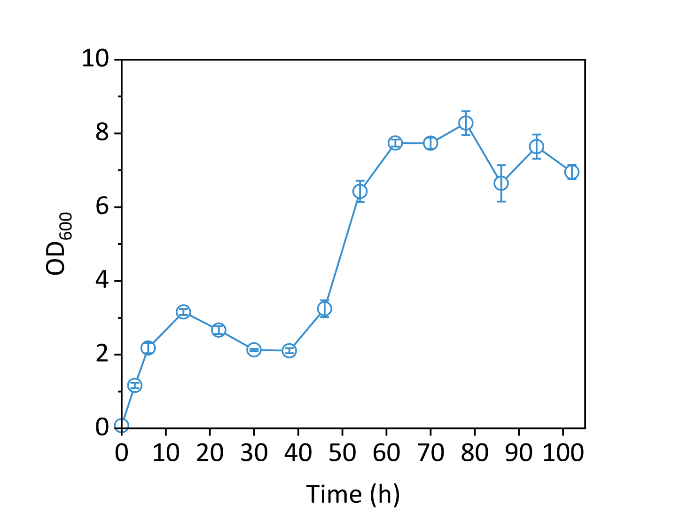


**Figure** **S6** Growth curve of strain *E*. *coli* Δ*thrA*Δ*psuT*Δ*argF*Δ*pepA*Δ*udk* pRSFDuet-1-*YjjG*-*psuG* pCDFDuet-1-*rihA*.


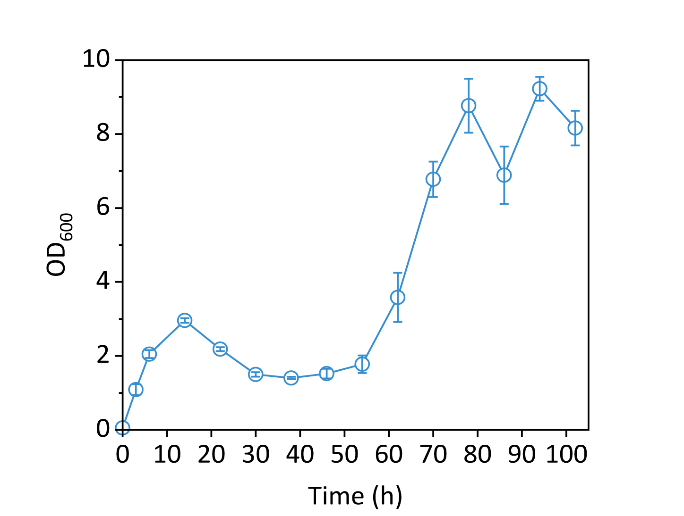


**Figure S7** Growth curve of *E*. *coli* Δ*thrA*Δ*psuT*Δ*argF*Δ*pepA*Δ*udk*Δ*udp*Δ*ppnp* pRSFDuet-1-*YjjG*-*psuG* pCDFDuet-1-*rihA*.


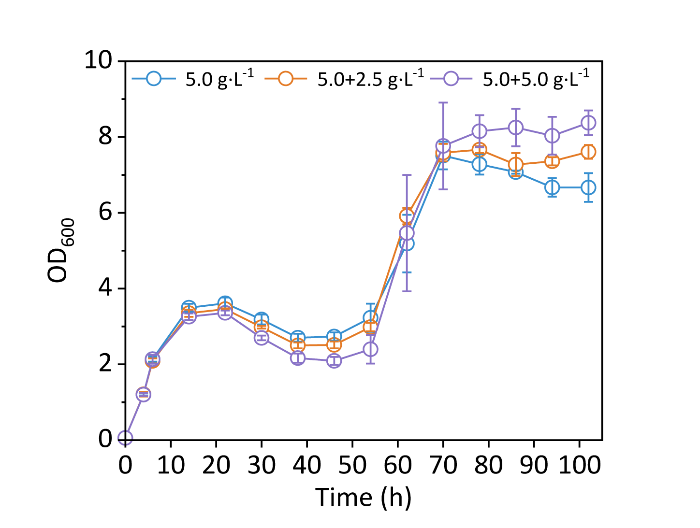


**Figure S8** Growth curve of *E*. *coli* Δ*thrA*Δ*psuT*Δ*argF*Δ*pepA*Δ*udk*Δ*udp*Δ*ppnp* pRSFDuet-1-*YjjG*-*psuG* pCDFDuet-1-*rihA*-*rbsK* via two-stage uridine supplementation strategy.


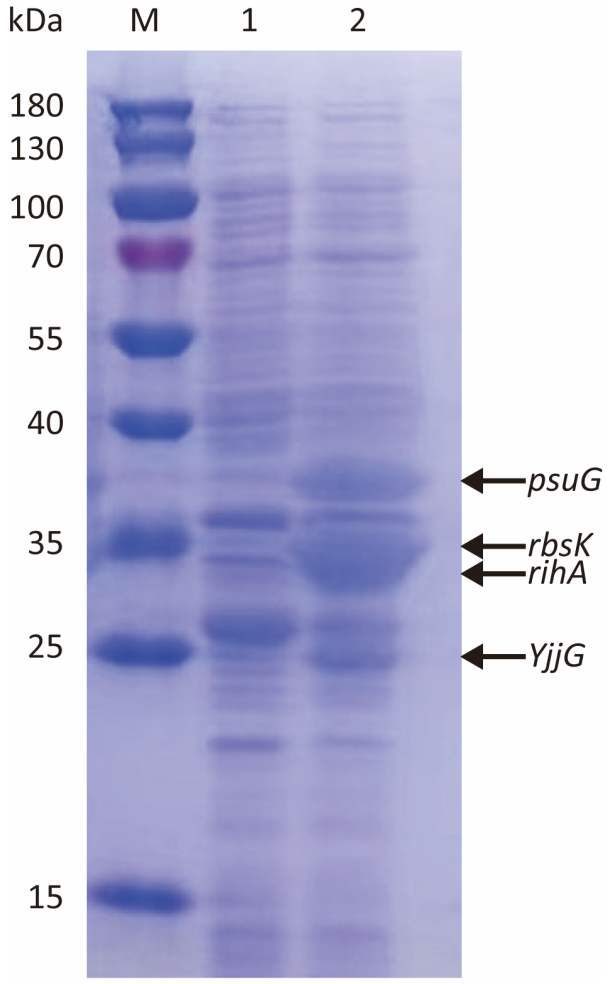


**Figure S9** SDS-PAGE of YjjG, PsuG, RihA, and RbsK in *E*. *coli* Δ*thrA*Δ*psuT*Δ*argF*Δ*pepA*Δ*udk*Δ*udp*Δ*ppnp* pRSFDuet-1-*YjjG*-*psuG* pCDFDuet-1-*rihA*-*rbsK*. M, Standard weight proteins (Maker); 1, *E*. *coli* Δ*thrA*Δ*psuT*Δ*argF*Δ*pepA*Δ*udk*Δ*udp*Δ*ppnp* (the control strain); 2, *E*. *coli* Δ*thrA*Δ*psuT*Δ*argF*Δ*pepA*Δ*udk*Δ*udp*Δ*ppnp* pRSFDuet-1-*YjjG*-*psuG* pCDFDuet-1-*rihA*-*rbsK*.


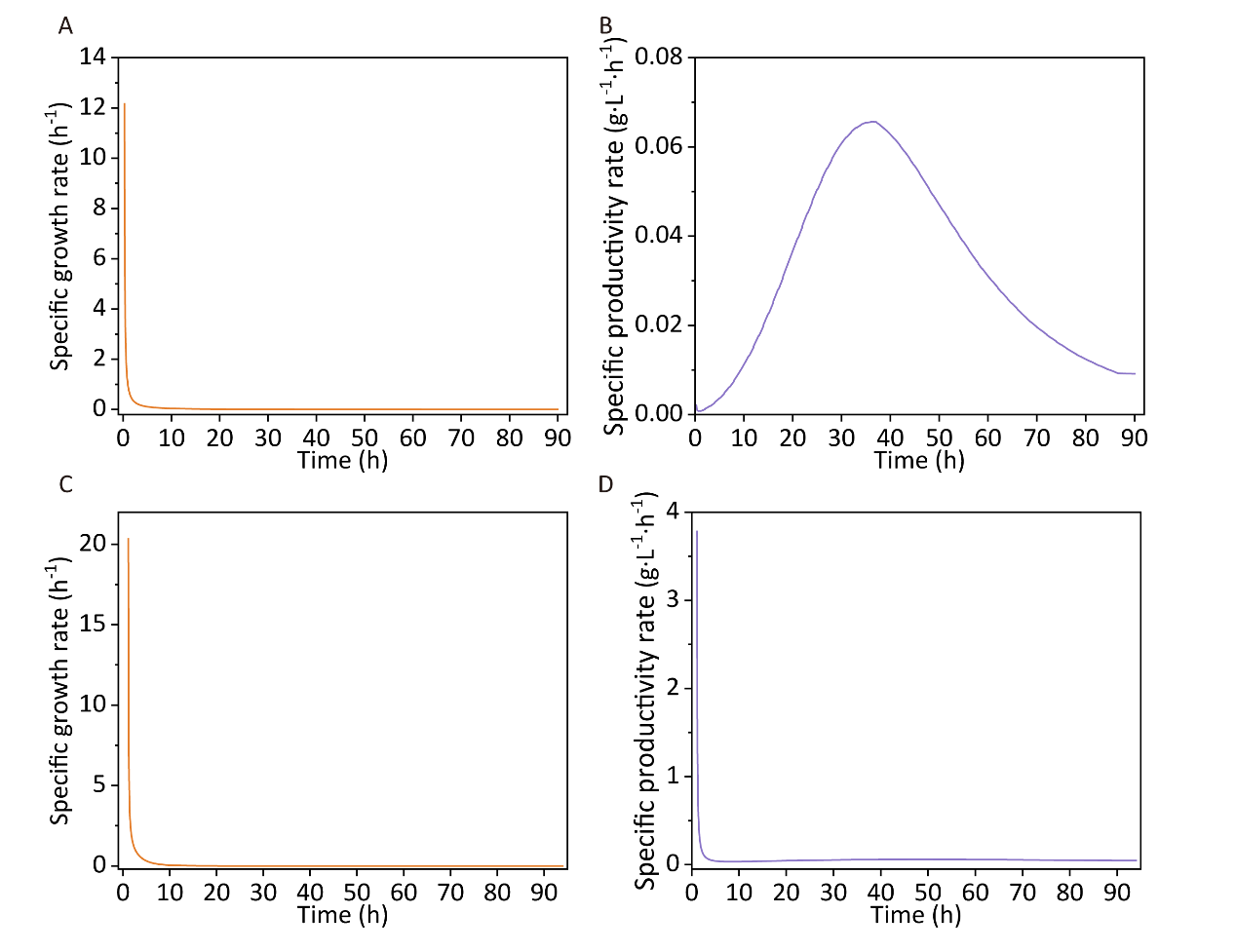


**Figure S10** Specific growth rate and specific production rate in a 5-L bioreactor. A, Specific growth rate in the 5-L bioreactor via supplementing 70 g·L^-1^ uridine. B, Specific production rate of pseudouridine in the 5-L bioreactor via supplementing 70 g·L^-1^ uridine. C, Specific growth rate in the 5-L bioreactor via supplementing 120 g·L^-1^ uridine. D, Specific production rate of pseudouridine in the 5-L bioreactor via supplementing 120 g·L^-1^ uridine.
